# Characterisation of the Faecal Bacterial Community in Adult and Elderly Horses Fed a High Fibre, High Oil or High Starch Diet Using 454 Pyrosequencing

**DOI:** 10.1371/journal.pone.0087424

**Published:** 2014-02-04

**Authors:** Kirsty Dougal, Gabriel de la Fuente, Patricia A. Harris, Susan E. Girdwood, Eric Pinloche, Raymond J. Geor, Brian D. Nielsen, Harold C. Schott, Sarah Elzinga, C. Jamie Newbold

**Affiliations:** 1 Institute of Biological Environmental and Rural Sciences, Aberystwyth University, Aberystwyth, Ceredigion, United Kingdom; 2 Equine Studies Group, WALTHAM Centre for Pet Nutrition, Melton Mowbray, Leicestershire, United Kingdom; 3 Michigan State University, Department of Animal Science, East Lansing, Michigan, United States of America; U. S. Salinity Lab, United States of America

## Abstract

Faecal samples were collected from seventeen animals, each fed three different diets (high fibre, high fibre with a starch rich supplement and high fibre with an oil rich supplement). DNA was extracted and the V1–V2 regions of 16SrDNA were 454-pyrosequenced to investigate the faecal microbiome of the horse. The effect of age was also considered by comparing mature (8 horses aged 5–12) versus elderly horses (9 horses aged 19–28). A reduction in diversity was found in the elderly horse group. Significant differences between diets were found at an OTU level (52 OTUs at corrected Q<0.1). The majority of differences found were related to the *Firmucutes* phylum (37) with some changes in *Bacteroidetes* (6), *Proteobacteria* (3), *Actinobacteria* (2) and Spirochaetes (1). For the forage only diet,with no added starch or oil, we found 30/2934 OTUs (accounting for 15.9% of sequences) present in all horses. However the core (i.e. present in all horses) associated with the oil rich supplemented diet was somewhat smaller (25/3029 OTUs, 10.3% ) and the core associated with the starch rich supplemented diet was even smaller (15/2884 OTUs, 5.4% ). The core associated with samples across all three diets was extremely small (6/5689 OTUs accounting for only 2.3% of sequences) and dominated by the order *Clostridiales*, with the most abundant family being *Lachnospiraceae*. In conclusion, forage based diets plus starch or oil rich complementary feeds were associated with differences in the faecal bacterial community compared with the forage alone. Further, as observed in people, ageing is associated with a reduction in bacterial diversity. However there was no change in the bacterial community structure in these healthy animals associated with age.

## Introduction

The gastrointestinal tract of mammals contains an extensive symbiotic microbial population composed of bacteria, phage, archaea, anaerobic fungi and protozoa. These microbes provide the host with essential nutrients, as well as modulating the immune system. In the horse, short chain fatty acids produced by microbial fermentation of fibre in the large intestine, provides a significant proportion of the animal’s daily energy requirements [1]. Despite the importance of microbes in supplying energy, little is still known about the overall composition of the microbial community (microbiome) in the equine hindgut. Several recent studies have characterised the faecal bacterial community of the equid using next generation sequencing [2]–[4]. However, none of these studies have investigated the effect of diet on the gut microbiota. Carnivores, herbivores and omnivores cluster according to differences in their gut microbiota [5] and diet is known to be a large driver of bacterial diversity between different species. In humans, diets enriched with animal protein and fat result in greater numbers of *Firmicutes* in the fecal microbiome, as compared to a plant based diets that are higher in fibre, that resultin higher numbers of *Bacteroidetes* and cellulose and xylan degraders [6]–[7]. Similarly, in mice fed high fat diets, *Firmicutes* (*Lachnospiraceae, Ruminococcus, Lactococcus*) numbers increased and *Bacteroidetes* numbers declined in fecal samples [8].

It has been well documented that sudden introduction of readily fermentable starch/sugar to a horse’s diet results in marked alterations in the colonic microbial ecosystem. Changes include a drop in pH and increases in *Streptococcus* spp. and lactate concentration with a subsequent increase in lactate-utilising bacteria [9]–[11]. Culture based microbiology has demonstrated increased counts of *Lactobacilli* and *Streptococci* coinciding with a decrease in cellulolytic bacterial numbers in horses maintained on a high starch diet compared to high fiber diet [12]–[13]. However, few studies have used culture-independent techniques to study the influence of diet on the bacterial population in healthy horses over time. Willing *et al*. [14] used terminal restriction fragment length polymorphism (TRFLP) to demonstrate a clear impact of high forage versus high carbohydrate diets on the fecal microbiome, where a high fibre diet resulted in greater stability and reduced bacteria associated with metabolic dysfunction. Whilst Daly *et al*. [15] found higher numbers of the phylum *Bacteroidetes*, the family *Lachnospiraceae* and the *Bacillus-Lactobacillus-Streptococcus* (BLS) group combined with lower numbers of *Fibrobacter* and *Ruminococcus* associated with increased dietary hydrolysable carbohydrate compared to a grass-based diet.

Age has also been shown to influence gastrointestinal bacterial diversity and community structure in humans. Studies have found an age-related reduction in diversity [16]–[17] with a decline in some *Bacteroidetes, Clostridia* and *Bifidibacteria* and an increase in *Proteobacteria* and *Bacilli* [17]–[19]. However, debate exists as to the influence of other environmental factors, including diet and geographical location [20]. To the authors’ knowledge, the influence of age has not been explored in relation to the bacterial community in the hindgut of the horse.

Here we present an investigation into the effect of three different diets (high fiber, fibre plus an oil rich supplement and fibre plus a starch rich supplement) on a group of seventeen healthy horses utilising 454 pyrosequencing to characterise the bacterial community of faeces. A cross over design was used allowing data to be collected from all 17 animals for each diet. Furthermore, the study group included 8 adult (5–12 yrs old) and 9 elderly individuals (19–28 yrs old).

## Materials and Methods

### Animal Nutrition Trial and Sample Collection

Faecal samples for microbiological investigation were collected as part of a wider nutritional study carried out at Michigan State University (USA). All animal procedures were approved by Michigan State University’s (MSU) Animal Care and Use Committee (approval #11/09-174-00). Seventeen healthy (with no dental abnormalities and that had recieved recent appropriate anthelmintic treatment) stock-type mares were chosen for their similar body type, size, body condition score (BCS) [21] and nutritional background (Table S1). The horses were classified as either adult (8 horses aged 5–12 yrs) or elderly (9 horses aged 19–28 yrs). Within each age group, horses were stratified by age and BCS in order to pair one adult and one aged horse together with one group of three containing the additional elderly horse. The study was a modified Latin Square, cross over design with horse pairs randomly assigned to one of three diets for each 6 week feeding period (Diet composition Table 1): hay alone (HAY), hay plus a high fibre, high oil and low cereal starch based complementary feed (OIL), and hay with a lower oil, cereal starch-rich concentrate (CHO). The hay used for all diets was timothy/mixed grass, the OIL diet was formulated with soya oil and the CHO diet was formulated using corn (cracked & crimped), oat pellets and soya oil. During the study period all horses received each of the three diets, therefore data collected for each diet was from 17 animals. The HAY diet was initially fed at 1.6% bodyweight (BW) but this was increased after 31 days to 1.84% BW in order to maintain BW. The OIL and CHO diets were fed initially at 0.6% BW combined with hay at 1.0% BW hay but this was increased to 0.69% BW concentrate and 1.15% hay 31 days into the study. All diets were divided between two daily feedings at 0800 and 2000 hours. Horses changing to the OIL and CHO diets were fed gradually increasing amounts adjusted over a four-day period. Throughout, the horses were allowed ad libitum access to water. During each dietary period, each pair was housed in an individual dry lot paddock for 3 weeks of outdoor feeding. Horses were moved to MSU’s Veterinary Teaching Hospital and housed individually in 2.4×3 m box stalls for the final 3 weeks of the feeding periods. While stalled, the horses were turned out in a dry lot paddock for a minimum of 1 hour three times a week.

**Table 1:** Diet composition of complete diets HAY (hay only), CHO (hay supplemented with starch), and OIL (hay supplemented with oil).

	HAY	CHO	OIL
**Energy (kcal/g)**	4.14	3.98	4.15
**NDF (%)**	61.4	25.0	42.3
**CP (%)**	7.9	13.2	14.9
**Fat (%)**	3.56	5.28	8.30
**Calcium (g/kg)**	7.9	11.4	15.9
**Phosphorus (g/kg)**	1.6	7.5	5.4
**Lignin (%)**	6.9	2.8	4.1
**WSC (%)**	10.6	7.6	8.6
**ESC (%)**	5.7	7.0	6.9
**Starch (%)**	0.5	35.2	5.4

Faecal samples were collected during the final day of each dietary period. Three samples were taken during each sampling day (early morning, mid-day & evening). Freshly voided faeces were selected and sub-sampled (approx 500 g) from the central portion to minimise contamination by bedding and flooring. After collection samples were stored on ice until frozen at −80°C prior to freeze drying.

### DNA Extraction

Prior to extraction of nucleic acids, freeze dried samples were disrupted by bead beating. Freeze- dried samples (100 mg) were added to a 2 ml screw top tube and one autoclaved glass ball was added (4 mm, undrilled, G/0300/53, Fisher Scientific, UK). Samples were beaten for 90 s at 5000 rpm (maximum speed) in a Mini-Beadbeater™ (Biospec products Inc., Bartlesville, OK). DNA was then extracted using QIAGEN QIAamp® DNA stool mini kits (Qiagen Ltd., UK) using the method described by Skřivanová *et al*. [22].

### PCR Amplification of 16S rDNA

Amplification of the V1–V2 hyper-variable regions of 16S rRNA was carried out with primers 27F and 357R [23]. The forward primer (5′-AGAGTTTGATCMTGGCTCAG-3′) carried the 454 Lib-L adaptor sequence B (5′-CCTATCCCCTGTGTGCCTTGGCAGTCTCAG-3′) and the reverse primer (5′-ACGAGTGCGTCTGCTGCCTYCCGTA-3′) carried the 454 Lib-L adaptor sequence A (5′-CCATCTCATCCCTGCGTGTCTCCGACTCAG-3′) followed by a 10 nucleotide sample specific barcode sequence (See Table S2). For each sample replicate PCR was performed in duplicate; a 25 µl reaction was prepared containing 5U µl^−1^ FastStart High Fidelity Enzyme Blend, 10x FastStart High Fidelity Buffer with 18 mM MgCl_2_(Roche Diagnostics Ltd., Burgess Hill, UK), 0.2 mM of each dNTP (Promega UK Ltd. Southampton, UK) with each primer used at 0.2 µM. For each reaction 1 µl DNA template at 2.5–125 ng/µl (as per Roche FastStart high Fidelity system recommendations) was used. The conditions used were a hot start of 95°C for 10 min, 95°C for 2 min followed by 22 cycles of 95°C for 30 s, 60°C for 30 s and 72°C for 45 s with a final extension at 72°C for 7 min. Reactions were amplified in a T100™ thermal cycler (Bio-Rad, Hemel Hempstead, UK). Resultant amplicons were visualized on a 1% (w/v) TAE agarose gel to assess quality of amplification before pooling the duplicate reactions.

### Short Fragment Removal and Pooling of Libraries and Sequencing

Pooled PCR reaction products for all sample replicates were purified as per Roche technical bulletin 2011-007 (January 2012) ‘Short Fragment Removal Procedure for the Amplicon Library Preparation Procedure’ using Agencourt AMpure XP beads (Beckman Coulter Inc.,Fullerton, USA). DNA concentration of the purified PCR products was assessed using an Epoch Microplate Spectrophotometer with a Take3 Micro-Volume plate (BioTek UK, Potton, UK) to enable equi-molar pooling of samples into four libraries each containing 36 to 39 samples with unique barcode sequences. Each library was further purified using the E-Gel® System with E-Gel® SizeSelect™ 2% Agarose gel (Life Technologies Ltd, Paisley, UK). A final purification step using Agencout AMpure XP beads standard PCR purification procedure (Beckman Coulter Inc.,Fullerton, USA) was carried out for each library. To assess purity of the sample libraries a quality control PCR was carried out for each as detailed in Roche technical bulletin 2011-007. 25 µl reactions were prepared containing: 5U µl^−1^ FastStart High Fidelity Enzyme Blend, 10x FastStart High Fidelity Buffer with 18 mM MgCl_2_ (Roche Diagnostics Ltd., Burgess Hill, UK), 0.2 mM of each dNTP (Promega UK Ltd. Southampton, UK) with each primer used at 0.2 µM. Primers used were the same as the Lib-L adapter sequences (described previously) as recommended in the Roche Technical Bulletin 2011-007. For each reaction 1 µl of each library containing 2×10^8^ molecules/µl was used. The conditions used were 94°C for 11 min followed by 20 cycles of 94°C for 1 min, 60°C for 1 min and 72C for 1 min with a final extension at 72°C for 10 min. On completion PCR products were incubated for 30 min at 37°C with 0.5 µl of Exonuclease I (New England BioLabs (UK) Ltd. Hitchin, UK). Reactions were amplified in a T100™ thermal cycler (Bio-Rad, Hemel Hempstead, UK). Products from the quality control PCR were assessed for quality and purified libraries were quantified on an Agilent 2100 Bioanalyzer with a High Sensitivity DNA chip (Agilent Technologies UK Ltd, Stockport, UK). The sample libraries were subsequently sequenced using the Roche 454 GS FLX Titanium series sequencer following ‘emPCR Method Manual-Lib-L’.

### Sequence Filtering, Processing and Statistical Analysis

Following sequencing data were combined and sample identification assigned to multiplexed reads using the MOTHUR software environment [24]. Data were denoised, low quality sequences, pyrosequencing errors and chimeras were removed then sequences were clustered into operational taxonomic units (OTU’s) at 97% identity using the CD-HIT-OTU pipeline (available from http://eeizhong-lab.ucsd.edu/cd-hit-otu [25]). OTU’s containing fewer than four reads per individual diet/animal combination were excluded due to the likelihood of them being a sequencing artifact. Samples were normalised by randomly resampling the sequences used to the lowest number of sequences per sample (each diet/animal combination) using Daisychopper (http://www.festinalente.me/bioinf/). Taxonomic classification of OTU’s was carried out using the Ribosomal Database Project (RDP) Classifier [26].

Data were prepared and tables and figures produced using Microsoft Excel and the ‘R’ software environment (version 2.15; http//www.r-project.org/). Simpson and Shannon-Wiener diversity indices were calculated using normalised data as recommended to reduce over-inflation of true diversity in pyrosequencing data sets [27]. Species richness and diversity were then analysed by two-way ANOVA using GenStat® 12^th^ edition. Each individual OTU was analysed for effects of diet and age by ANOVA (using GenStat® 12^th^ edition). Although it was not possible to calculate skewness of the data, due to having only one replicate per treatment/animal combination, skewness was considered across all animal/treatment combinations for each OTU. Many of the OTUs showed marked skewness. Nevertheless ANOVA was performed as there is no non-parametric equivalent for a multi-factorial experiment. However to minimise the false discovery rate only OTUs with more than 100 occurances (across all samples) were analysed by ANOVA (579 OTUs). Furthermore, P values were adjusted using the method of Benjamini and Hochberg [28] where significance was set at Q <0.1. A phylogenetic tree was constructed to display OTUs found to be significant for diet. The tree was constructed by 1) Sequences were aligned using the Ribosomal Database Project (RDP) Pyrosequencing pipeline Aligner which utilizes the Infernal aligner [29] 2) Tree built by UPGMA using Molecular Evolutionary Genetic Analysis tool (MEGA) version 5.2. [30] 3) Tree was graphically manipulated using the interactive tree of life (iTOL) tool [31]. The core community at OTU level in faeces was defined by being present in all samples (each animal/diet combination) included in the study. When considering the core community in faeces for each diet, it was defined by being present in all animals for each diet.

### Nucleotide Sequence Accession Numbers

16S rDNA sequences were deposited with the European Nucleotide Archive under study accession number: PRJEB4523 found at http://www.ebi.ac.uk/ena/data/view/PRJEB4523.

## Results

All horses remained healthy throughout the study with no gastrointestinal disturbances. One million, four hundred and sixty thousand, one hundred and twenty four sequences of average length 358 bp were obtained from the 454 FLX Titanium sequencing. Quality filtering resulted in 749,437 high quality sequences that were clustered into 5689 unique OTUs. Construction of a phylogenetic tree (data not shown) indicated that all samples from an animal on a sampling day (three across the day) clustered tightly together which allowed data from these samples to be pooled providing a minimum of 6197 sequences per sample day after normalisation. Rarefaction curves calculated from non-normalised data (Figure S1) showed that for each sample the corresponding curve had not plateaued indicating that complete sampling of these environments had not yet been achieved. Good’s coverage estimates, however, indicated that a large part of the diversity in all samples had been captured with the average coverage being 96.3% (s.d. 1.6).

**Figure 1:**
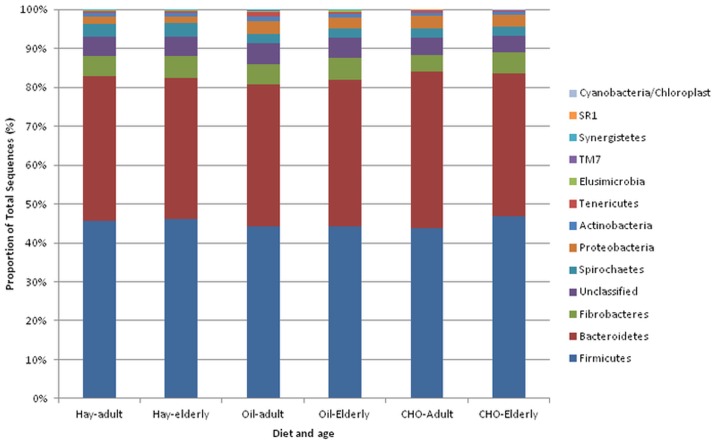
Phyla identified and relative proportion of each associated with different diets and age of horse. Data shown from the bacteria community in faeces from horses fed three diets; Hay- high fibre diet, Oil- high oil diet, CHO- high starch diet. Horses fed these diets were also assigned to two age groups adult or elderly.

**Figure 2:**
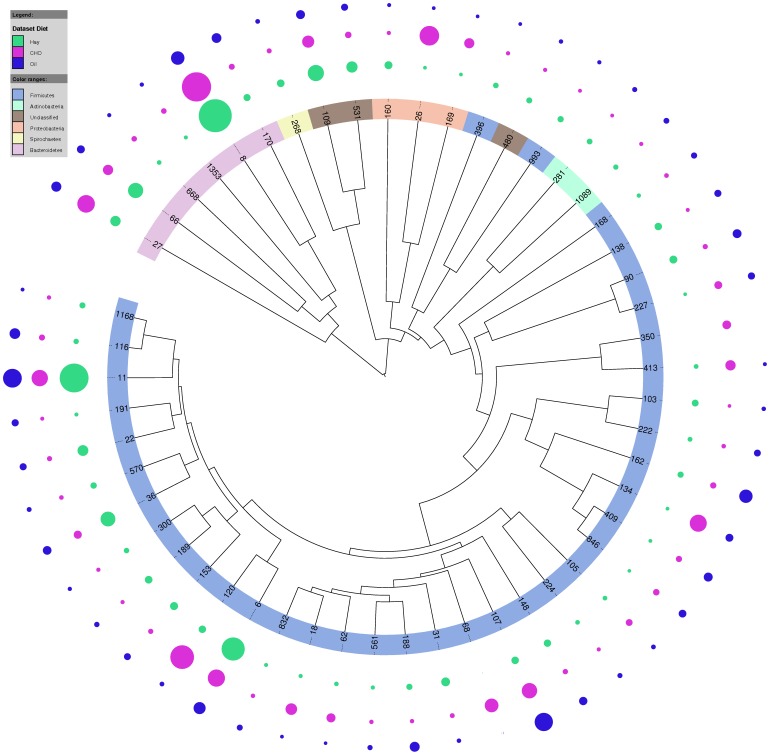
Phylogenetic tree showing significant OTUs (corrected P<0.1) for diet. Tree shows only those OTUs found to be significant (corrected P<0.1) and was built using UPGMA. The coloured outer ring indicates the bacterial phyla each OTU belongs to while the outer circles show the relative abundances of each OTU for the different diets; 1 layer of circles for Hay- high fibre diet,1 for Oil- high oil diet and 1 for CHO- high starch diet.

The bacterial community within faeces of horses on different diets was found to be highly diverse and even (as indicated by the Simpson and Shannon-Weiner diversity indices, Table 2) with no significant differences between diets. The bacterial community was significantly less diverse in the elderly horses (Shannon-Wiener P = 0.018, species richness P = 0.042). When exploring the data by principle component analysis (Figure S2) no pattern was observed according to either diet or age. As principle component 1 (PC1) and principle component 2 (PC2) only accounted for 25% of the variance within the data discriminant function analysis with cross validation was conducted, however the model would not validate. Little difference could similarly be seen in the relative abundance of bacterial phyla under different diet/age combinations (Figure 1) with the only notable difference being an increase in *Proteobacteria* in the high oil and high starch diets.For all diet/age combinations the bacterial population in faeces was dominated by the *Firmicutes* (45%) followed by the *Bacteroidetes* (37%) with smaller quantities (0.5–3.5%) of *Proteobacteria, Spirochaetes, Actinobacteria* and *Tenericutes* and very small amounts (0–0.4%) of *Elusimicrobia, TM7, Synergistetes, SR1* and *Cyanobacteria/Chloroplast*. Values shown for each phyla show little variation between individual animals (Table S3). Although little difference was seen at Phyla level when each individual OTU was compared by ANOVA, 52 were significantly different between diets but none were significantly different between age groups (corrected P<0.1). For the OTUs that differed according to diet, 37 were *Firmicutes*, 6 *Bacteroidetes,* 3 *Proteobacteria*, 3 *Unclassified,* 2 *Actinobacteria* and 1 *Spirochaetes*. A comparison of how the three diets varied specific to these OTUs can be seen in Figure 2. The relative abundance of each OTU significant for diet is shown in Table S4 with classification of these OTUs to genus level in Table S5.

**Table 2:** Diversity and Richness of the microbial communities in faeces of horses from two different age groups (adult or elderly) and receiving three different diets (Hay, CHO, Oil).

	Diet				Age			Diet x Age
	Hay	CHO	Oil	P Value	Adult	Elderly	P Value (S.E.D.)	P Value
**Species Richness**	671	623	698	P = 0.105(34.6)	727^b^	601^a^	P = 0.042 (54.6)	P = 0.83
**Simpson’s Diversity**	0.992	0.989	0.989	P = 0.559(0.003)	0.992	0.988	P = 0.074(0.002)	P = 0.56
**Shannon-Wiener Diversity**	5.758	5.576	5.785	P = 0.066(0.092)	5.831^b^	5.582^a^	P = 0.018 (0.0895)	P = 0.95

Different superscript letters denote significant differences.

**Figure 3:**
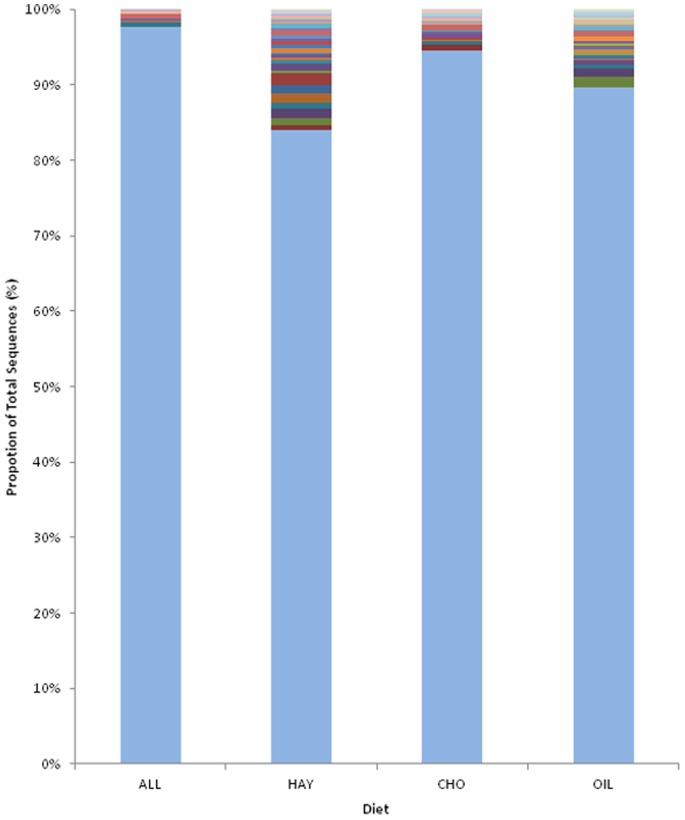
The core bacterial community associated with different diets and age of horse. The core community for All is defined as those OTUs (clustered at 97% similarity) present in all animals for all diets and which abundances are 0.1% (or greater) of the total number of sequences. The core for each of the three diets is defined as those OTUs (clustered at 97% similarity) present in all samples from each individual diet and which abundances are 0.1% (or greater) of the total number of sequences for each diet. The lower pale blue section of the bar indicates the proportion that is not part of the core. The remaining individual coloured sections represent each OTU of which the core is comprised; All (6), HAY(30), CHO (15), OIL(25). For details of classification and abundance of individual OTU’s see table S6.

A small number of OTUs were identified which made up the core community in all faecal samples (all diets), 6 OTUs accounting for 2.3% of total sequences (Figure 3). When identifying a core community for each individual diet, a larger core was found than across all three diets; HAY diet-30 OTUs accounting for 15.9%, OIL diet-25 OTUs accounting for 10.3%, and CHO diet-15 OTUs accounting for 5.42%. When classified to family level (Table S5), it can be seen that the core community found in all diets is dominated by the order *Clostridiales (Lachnospiraceae, Clostridiales_Incertae Sedis XIII* & *Ruminococcaceae)* with one unclassified *Bacteroidetes.* For each diet, when selecting only families accounting for over 1% of the total sequences, all were dominated by *Lachnospiraceae* but diet specific differences were clear with the remainder. For the hay diet, this included *Porphyromonadaceae*, *Fibrobacteraceae, Unclassified Clostridia* and *Prevotellaceae,* with *Fibrobacteraceae* not identified as part of the core community on either of the other diets. For the CHO diet, only the *Lachnospiraceae* were found at 1% or greater with no families identified that appear to be unique to the core community for this diet. Lastly, the OIL diet, similar to the HAY diet, included *Porphyromonadaceae* as the second largest component, followed by an unclassified *Firmicutes* then an unclassified *Bacteroidetes.* Other than these more abundant members of the core community associated with the oil diet, there were two families not identified as part of the core of the other two diets*; Spirochaetaceae* and a *Proteobacteria* order *Rhizobiales.*

## Discussion

The horse is reliant on intake of large quantities of fibre that can be fermented by the microbiota found predominantly within the large intestine, to yield short chain fatty acids which in turn can be utilised for energy by the host. Due to increased demands placed on the equid for athletic performance, modern nutritional practices involve supplementation of this fibre-based diet with varying quantities of high energy-providing feedstuffs, in particular cereal grains and oil. When cereal starch is fed in small quantities, it is subjected to enzymatic breakdown and absorption of sugars in the small intestine, however when fed in large quantities, the small intestine’s capacity for starch digestion can be overloaded and a considerable amount may be passed to the hindgut where it undergoes rapid microbial fermentation [10]–[11], [32]. To accurately document how different diets influence the bacterial community in the caecum and colon, direct sampling following controlled feeding trials would be ideal. However, this is not practical and involves either cannulation or euthanasia. A more practical alternative is to document changes in the hindgut through exploration of the faecal microbiota which might be expected to contain representative organisms from all regions of the large intestine as they are moved physically with the digesta. In human studies, it has been suggested that faecal samples do not accurately reflect the population of the rest of the intestinal tract [33]–[34]. Our previous work has demonstrated that there are differences in the microbial community found in the caecum compared to the right dorsal colon and faeces of horses and ponies with the main change in composition occurring at the point of the pelvic flexure between the ventral and dorsal colon. However, the microbiota of the distal hindgut (right dorsal colon through to the rectum) shares a similar composition to that of faeces [35]–[36], which, considering the importance of the right dorsal colon [37] in fibre fermentation, means that studying the microbiota of faeces can provide useful information.

From the limited published work which has utilised next generation sequencing to document the bacterial community in the large intestine of the horse [2]–[4], the dominant phylum appears to be *Firmicutes* (43–69%). We have similarly found this both in our previous work (46% [36]) and in the current study regardless of diet or age (average 45%). There is, however, inconsistency over the next most abundant phylum (*Bacteroidetes* 14.2%, *Proteobacteria* 10.2% [2], *Proteobacteria* and *Verrucomicrobia* 4.1% each, *Bacteroidetes* 3.65% [3] or *Verrucomicrobia* 18.1%, *Bacteroidetes* 5.7% [4]). The higher numbers of *Bacteroidetes* found in both this study (average 37%) and our previous work (43%) is in agreement with older culture-independent work [14], [38]. We have identified smaller quantities (<5%) of *Fibrobacteres, Proteobacteria*, *Spirochaetes* and *Actinobacteria*, again regardless of diet or age which is in agreement with our previous work and that of others [3]–[4], [36] with a notable lack of *Verrucomicrobia* [2].

The concept of a core microbial population in the gut of mammals has recently received attention with a core community in the human gut identified, but differing in size dependent on the study design [39]–[41] . What constitutes a core microbiome has not been well defined and factors such as sequencing depth and OTU clustering methods may influence identification of the presence of a core, estimation of the true size of that core (both the number and relative abundance of OTUs) and detection of core members [40]–[42]. In the horse, a core bacterial community in faeces has been suggested; with 123 out of 1620 identified OTUs present in each of 4 animals, with 6 of these having an abundance of greater than 25 occurrences per animal [2]. Our previous work [36] also identifies a core, but smaller in size (25/2566 OTUs accounting for 13.7% of sequences). When considering the core community associated with a forage only, HAY diet in the current study, a similar size of core is seen to our previous work (30/2934 OTUs accounting for 15.9% of sequences). However, the core associated with the OIL diet was somewhat smaller (25/3029 OTUs accounting for 10.3% of sequences) and the core associated with a high starch providing, CHO diet was much smaller (15/2884 OTUs accounting for 5.4% of sequences). Interestingly, the core associated with samples across all three diets is extremely small in comparison to both the individual diets (6/5689 OTUs accounting for 2.3% of sequences) and other core communities from other gut environments such as in the rumen of the cow (157/4986 OTUs) [43]. Another feature of the core community in the horse is that it is comprised of low abundance OTUs and is not dominated by any individual OTUs or bacterial families; the largest family found in the core associated with all diets is responsible for only 1.3% of all sequences. Core populations in other environments such as the oral cavity of dogs [44] and humans [45] and the tonsils of pigs [46] are characterised by having a few highly dominant OTUs. Regardless of the approach used, clostridia have been consistently identified as the most prevalent class of bacteria in the core community of the human gut [39]–[40], [42], [47]–[48]. Members of this class that have been identified as part of the human core are *Ruminococcaceae, Lachnospiraceae, Clostridiaceae* and *Streptococcaceae* [39]–[40], [42], [48]. Bacteroidetes have also been shown to be core, but at low numbers [42], [48]. Our data for the horse would interestingly seem to mirror this pattern with *Lachnospiraceae* being the most abundant in the core in the current work (also found by Costa *et al*. [2]). Our previous data [36] identified *Prevotellaceae* followed by *Ruminoccocaceae*, *Fibrobacteraceae,* then *Lachnospiraceae* as being the most abundant members of the core community and is similar to what has been reported in cattle [43], [49]–[50]. As *Lachnospiraceae,* in particular, have been shown to exist in most mammals [39]–[40], [42] and have been found in 71% of the order Perissodactyla [51], this would be suggestive that this family may appear in the intestinal core of all mammals. The importance of this family in a gut core bacterial population is perhaps not surprising given that *Lachnospiraceae* are known butyrate producers [42], [52] and butyrate is known to have a protective function on colonocytes in the gut wall [53]–[54].

The lack of a substantial bacterial core combined with a lack of obvious ‘key’ members may help explain why the horse is so susceptible to disruption in its microbiota from its normal state resulting in subsequent gastrointestinal disorder [36]. Furthermore, the reduction in core size when horses are fed a diet other than one high in fibre and, particularly when fed a high starch supplemented diet, may increase the risk for subsequent metabolic dysfunction.

The gastrointestinal tract of humans is initially colonised by bacteria at birth and the population increases in diversity over the first three years of life [55]. As the individual moves into adulthood, the composition remains relatively stable over time providing there is no disruption such as disease or antibiotic usage [42], [56]. It is widely acknowledged that as humans become elderly, the microbial population in their gastrointestinal tract changes and, in particular, bacterial diversity declines [16]–[17], [57]. Reduction in diversity may similarly be attributed to physiological changes associated with ageing such as increased digesta transit time and a reduced requirement for dietary energy [58]. However, elderly people are often undergoing drug treatment regimens to support a range of conditions and the effects of these drugs on the gut bacteria are not always known [59]. Differences between studies in the proportion of *Bacteroidetes* found in the elderly may result from these confounding factors as an increase has been reported in some cases [57], [59] but others have seen a decrease [18], [20]. A decline in Clostridia cluster XIV [17]–[18], [20] and cluster IV [18], [60] has been reported. While other bacteria shown to change with age include *Bifidobacteria* (decline), *Proteobacteria* and *Bacilli* (increase) [17], [19], [57], [60]. Here we report a reduction in diversity in the aged animals similar to that demonstrated in humans, althougth no significant differences in individual species of bacteria were found and in the larger study relating to our group of horses no difference in digestibility was found between the adult and elderly horses [61].

In other mammals (human, mouse, cow), diet has been shown to be a strong driver of gut microbiota with obvious clustering of individuals according to type of diet [6]–[8], [50]. In this study, such a pattern was not seen when analysing the total bacterial population, which is in a contrast with recent work in cows where samples clustered separately when starch was added to the diet [62]. Only when individual OTUs were analysed were differences found, a possible explanation for this may be that as the core bacterial community in the faeces of the horse accounts for only a small proportion of the total community, changes may not be big enough to be seen at a general level of investigation. Furthermore, the effect of individual animals would appear to have a highly significant effect on the bacterial community and may indeed mask any dietary effects [14]. Of the 52 OTUs found to show significant differences between diets (corrected P<0.1), the majority are classified as *Firmicutes* (37 OTUs) with some *Bacteroidetes* (6 OTUs), *Proteobacteria* (3 OTUs), *Actinobacteria* (2 OTUs) and Spirochaetes (1 OTU). Different OTUs belonging to the family *Lachnospiraceae* were found at different numbers in each of the three diets, with specific OTUs belonging to this family being significantly higher for each diet. When feeding the forage only diet, the OTUs that are significantly more abundant than in the other two diets are primarily *Clostridiales*. For the forage only and OIL diets, a member of the *Firmicutes* phylum, genus *Acidaminococcus* was significantly more abundant than with the starch supplemented diet. The OIL diet also shows most significant increases in abundance associated with the *Clostridiales*, including elevated numbers of *Ruminococcus Oscillibacter*. Although little is known about the function of this genus, it has been found to increase in abundance associated with high fat diets of mice [63] and humans [64]; indicating a role in fat metabolism. When considering the starch supplemented diet, yet again the biggest changes seen centre around the *Clostridiales*, including *Lachnospiraceae,* similar to the influence of starch in cattle [62], and *Roseburia.* Also of note is an increase in abundance of an OTU belonging to the *Proteobacteria* phylum, genus *Succinivibrio*. Both *Roseburia* and *Succinivibrio* have been shown to significantly increase in cattle changed from a high fibre to a high starch diet [50]. *Roseburia* is a butyrate producer and although little is known about the function of *Succinivibrio,* it is found to be more abundant in cattle on a high starch diet [50], [65] and it has also been identified as an important component of the gut microbiome in the bee which relies on a starch rich diet [66]. Enrichment of *Succinivibrio* in these gut environments may be suggestive of a role in starch metabolism. Previous work in relation to dietary change in the horse has identified increases in *Lachnospiraceae* and the *Bacteroidetes* phylum associated with a high starch diet [15]. The fact that the *Clostridiales* appear to be the most influential across all diets in this study is consistent with findings in humans that the Clostridia Clusters IV and XIVa are very sensitive to dietary influence [59].

## Conclusions

Characterisation of the bacterial community from the hind gut of healthy horses is essential to enable comparison to disease state and thus develop diagnostic tests, prophylactic measures and appropriate treatments. Here we show that feeding different diets results in significant changes in the faecal bacterial microbiome. Also identified is a reduction in bacterial diversity in older horses. Furthermore we confirm the presence of only a small core bacterial community which is found in all horses regardless of age or diet, composed predominantly of the *Lachnospiraceae.* The presence of such a small core may begin to explain why the horse is so susceptible to metabolic dysfunction.

## Acknowledgments

The authors would like to thank the late Dr David Causton for his invaluable advice regarding statistical analysis of this data. (IBERS, Aberystwyth University).

## Supporting Information

Figure S1 Rarefaction Curves showing depth of sequencing of the microbial communities of faeces from eighteen horses fed three different diets (Calculated from non-normalised data).
(DOCX)

Figure S2 Principle component analysis of relative abundance of OTUs identified from faceal samples from eighteen horses fed three different diets. Samples are coloured by age/diet combinations (Hay diet adult, hay diet elderly, fat diet adult, fat diet elderly, starch diet adult, starch diet elderly).
(DOCX)

Table S1 Animal metadata.
(DOC)

Table S2 MID barcode sequences used for multiplexed 454 pyrosequencing.
(DOCX)

Table S3 Relative abundance of different bacterial phyla found in the faeces of horses fed three different diets.
(DOCX)

Table S4 Relative abundance of each OTU significant (P<0.001) for Diet, Age or Diet*Age.
(DOCX)

Table S5 Classification of each OTU significant (P<0.001) for Diet, Age or Diet*Age.
(DOCX)

Table S6 Classification of the core bacterial community in the faeces of horses fed three different diets.
(DOCX)
