# Supplementary material for: Characterisation of the Faecal Bacterial Community in Adult and Elderly Horses Fed a High Fibre, High Oil or High Starch Diet Using 454 Pyrosequencing
Source: PLoS One. 2014 Feb 4;9(2):e87424. doi: 10.1371/journal.pone.0087424 (PMC3913607; doi:10.1371/journal.pone.0087424)

**Figure S1**. Rarefaction Curves showing depth of sequencing of the microbial communities of faeces from eighteen horses fed three different diets (Calculated from non-normalised data)


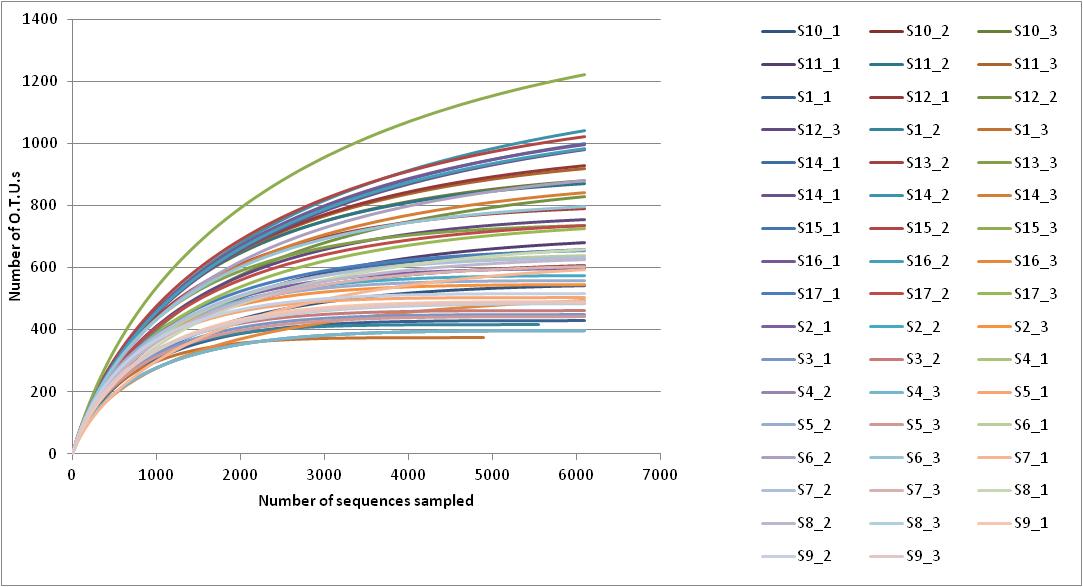

Supplement: Figure S1 — Rarefaction Curves showing depth of sequencing of the microbial communities of faeces from eighteen horses fed three different diets (Calculated from non-normalised data). (DOCX) [file pone.0087424.s001.docx]
