# Supplementary material for: Characterisation of the Faecal Bacterial Community in Adult and Elderly Horses Fed a High Fibre, High Oil or High Starch Diet Using 454 Pyrosequencing
Source: PLoS One. 2014 Feb 4;9(2):e87424. doi: 10.1371/journal.pone.0087424 (PMC3913607; doi:10.1371/journal.pone.0087424)

**Figure S2**-Principle component analysis of relative abundance of OTUs identified from faceal samples from eighteen horses fed three different diets. Samples are coloured by age/diet combinations (Hay diet adult, hay diet elderly, fat diet adult, fat diet elderly, starch diet adult, starch diet elderly).


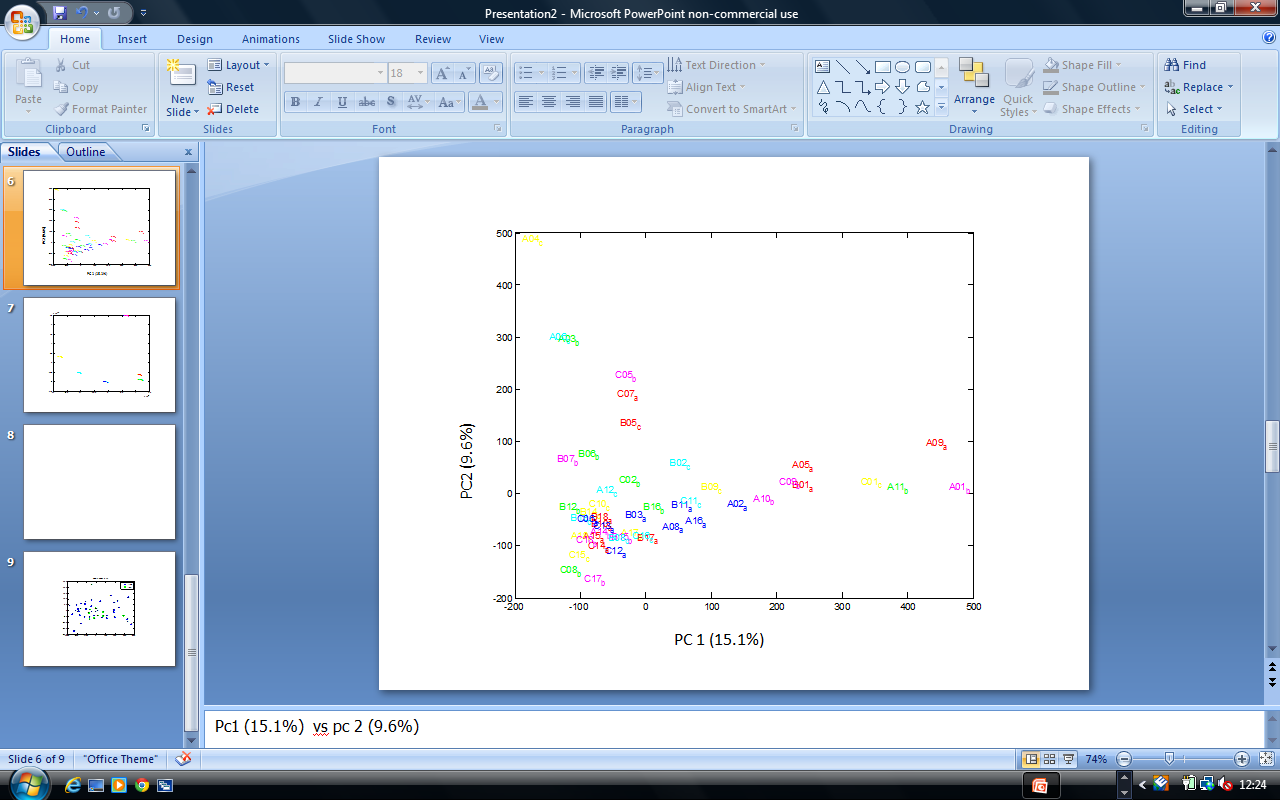

Supplement: Figure S2 — Principle component analysis of relative abundance of OTUs identified from faceal samples from eighteen horses fed three different diets. Samples are coloured by age/diet combinations (Hay diet adult, hay diet elderly, fat diet adult, fat diet elderly, starch diet adult, starch diet elderly). (DOCX) [file pone.0087424.s002.docx]
