# Supplementary material for: Characterisation of the Faecal Bacterial Community in Adult and Elderly Horses Fed a High Fibre, High Oil or High Starch Diet Using 454 Pyrosequencing
Source: PLoS One. 2014 Feb 4;9(2):e87424. doi: 10.1371/journal.pone.0087424 (PMC3913607; doi:10.1371/journal.pone.0087424)
Supplement: Table S1 — Animal metadata. (DOC) [file pone.0087424.s003.doc]

**Table S1**. Animal metadata

| Animal number | Age | Breed | Bodyweight at start | Body condition score at start |
| --- | --- | --- | --- | --- |
| 1 | 20* | Stock-type | 474 | 5.3 |
| 2 | 20* | Stock-type | 491 | 5.0 |
| 3 | 10* | Stock-type | 550 | 5.5 |
| 4 | 20* | Stock-type | 461 | 5.0 |
| 5 | 11* | Stock-type | 499 | 5.5 |
| 6 | 10* | Stock-type | 514 | 5.0 |
| 7 | 20* | Stock-type | 464 | 5.0 |
| 8 | 4* | Thoroughbred | 511 | 4.0 |
| 9 | 30 | Stock-type | 461 | 5.5 |
| 10 | 20* | Stock-type | 478 | 4.5 |
| 11 | 11* | Stock-type | 544 | 5.5 |
| 12 | 10* | Stock-type | 450 | 4.5 |
| 13 | 20* | Stock-type | 434 | 5.0 |
| 14 | 19 | Thoroughbred | 523 | 5.0 |
| 15 | 10* | Stock-type | 500 | 5.5 |
| 16 | 20* | Stock-type | 425 | 5.0 |
| 17 | 8 | Thoroughbred | 470 | 4.5 |

* Age estimated by Veterinary dental examination.
