# Supplementary material for: Characterisation of the Faecal Bacterial Community in Adult and Elderly Horses Fed a High Fibre, High Oil or High Starch Diet Using 454 Pyrosequencing
Source: PLoS One. 2014 Feb 4;9(2):e87424. doi: 10.1371/journal.pone.0087424 (PMC3913607; doi:10.1371/journal.pone.0087424)
Supplement: Table S3 — Relative abundance of different bacterial phyla found in the faeces of horses fed three different diets. (DOCX) [file pone.0087424.s005.docx]

**Table S3**.Relative abundance of different bacterial phyla found in the faeces of horses fed three different diets.

| **Bacterial Phyla** | **Hay-adult** | | **Hay-elderly** | | **Fat-adult** | | **Fat-Elderly** | | **CHO-Adult** | | **CHO-Elderly** | |
| --- | --- | --- | --- | --- | --- | --- | --- | --- | --- | --- | --- | --- |
|  | **Mean** | **St-dev** | **Mean** | **St-dev** | **Mean** | **St-dev** | **Mean** | **St-dev** | **Mean** | **St-dev** | **Mean** | **St-dev** |
| Firmicutes | 45.75 | 0.45 | 46.08 | 0.95 | 44.18 | 1.12 | 44.30 | 0.85 | 43.76 | 1.53 | 46.83 | 1.12 |
| Bacteroidetes | 37.06 | 0.49 | 36.33 | 0.63 | 36.54 | 0.89 | 37.60 | 0.50 | 40.23 | 1.00 | 36.63 | 0.70 |
| Fibrobacteres | 5.20 | 0.14 | 5.55 | 0.52 | 5.31 | 0.65 | 5.79 | 0.86 | 4.19 | 0.54 | 5.57 | 0.45 |
| Unclassified | 5.06 | 0.65 | 5.05 | 0.13 | 5.43 | 0.18 | 5.14 | 0.15 | 4.67 | 0.30 | 4.18 | 0.16 |
| Spirochaetes | 3.31 | 0.13 | 3.49 | 0.19 | 2.37 | 0.19 | 2.40 | 0.10 | 2.31 | 0.19 | 2.30 | 0.14 |
| Proteobacteria | 1.76 | 0.09 | 1.75 | 0.11 | 3.28 | 0.30 | 2.62 | 0.18 | 3.22 | 0.26 | 3.22 | 0.22 |
| Actinobacteria | 0.88 | 0.10 | 0.86 | 0.05 | 1.01 | 0.05 | 0.93 | 0.03 | 0.65 | 0.03 | 0.56 | 0.04 |
| Tenericutes | 0.60 | 0.05 | 0.55 | 0.09 | 1.37 | 0.19 | 0.63 | 0.04 | 0.52 | 0.08 | 0.25 | 0.03 |
| Elusimicrobia | 0.18 | 0.01 | 0.15 | 0.02 | 0.17 | 0.02 | 0.40 | 0.05 | 0.09 | 0.01 | 0.08 | 0.01 |
| TM7 | 0.14 | 0.01 | 0.13 | 0.01 | 0.24 | 0.04 | 0.13 | 0.02 | 0.20 | 0.03 | 0.19 | 0.02 |
| Synergistetes | 0.04 | 0.00 | 0.04 | 0.01 | 0.06 | 0.01 | 0.04 | 0.01 | 0.09 | 0.02 | 0.05 | 0.01 |
| SR1 | 0.02 | 0.00 | 0.02 | 0.00 | 0.03 | 0.01 | 0.02 | 0.01 | 0.07 | 0.03 | 0.02 | 0.00 |
| Cyanobacteria/Chloroplast | 0.01 | 0.00 | 0.02 | 0.02 | 0.01 | 0.00 | 0.00 | 0.00 | 0.00 | 0.00 | 0.11 | 0.02 |
