# Supplementary material for: Characterisation of the Faecal Bacterial Community in Adult and Elderly Horses Fed a High Fibre, High Oil or High Starch Diet Using 454 Pyrosequencing
Source: PLoS One. 2014 Feb 4;9(2):e87424. doi: 10.1371/journal.pone.0087424 (PMC3913607; doi:10.1371/journal.pone.0087424)
Supplement: Table S4 — Relative abundance of each OTU significant (P<0.001) for Diet, Age or Diet*Age. (DOCX) [file pone.0087424.s006.docx]

**Table S4** Relative abundance of each OTU significant (P<0.001) for Diet, Age or Diet*Age

|  | **HAY** | | **CHO** | | **OIL** | | **SED** | | |
| --- | --- | --- | --- | --- | --- | --- | --- | --- | --- |
| **O.T.U.** | **Adult** | **Elderly** | **Adult** | **Elderly** | **Adult** | **Elderly** | **Diet** | **Age** | **Diet*Age** |
| 6 | 0.773 | 0.789 | 0.539 | 0.548 | 0.333 | 0.345 | 0.114** | 0.090 | 0.231 |
| 8 | 1.183 | 1.158 | 0.943 | 1.097 | 0.323 | 0.466 | 0.221** | 0.232 | 0.345 |
| 11 | 0.786 | 1.226 | 0.476 | 0.544 | 0.494 | 0.719 | 0.145** | 0.180 | 0.246 |
| 18 | 0.020 | 0.026 | 0.370 | 0.269 | 0.000 | 0.019 | 0.048*** | 0.041 | 0.069 |
| 22 | 0.225 | 0.355 | 0.034 | 0.092 | 0.159 | 0.120 | 0.038*** | 0.047 | 0.064 |
| 26 | 0.000 | 0.017 | 0.575 | 0.700 | 0.000 | 0.014 | 0.125*** | 0.098 | 0.174 |
| 27 | 0.280 | 0.262 | 0.500 | 0.617 | 0.158 | 0.361 | 0.093** | 0.090 | 0.140 |
| 31 | 0.222 | 0.198 | 0.103 | 0.003 | 0.051 | 0.021 | 0.055** | 0.080 | 0.102 |
| 36 | 0.299 | 0.597 | 0.192 | 0.169 | 0.119 | 0.288 | 0.053*** | 0.080 | 0.101 |
| 62 | 0.028 | 0.051 | 0.132 | 0.304 | 0.008 | 0.000 | 0.052*** | 0.037 | 0.070 |
| 66 | 0.399 | 0.528 | 0.286 | 0.247 | 0.208 | 0.140 | 0.084** | 0.126 | 0.160 |
| 68 | 0.001 | 0.000 | 0.299 | 0.512 | 0.001 | 0.000 | 0.117** | 0.092 | 0.164 |
| 90 | 0.165 | 0.182 | 0.072 | 0.063 | 0.272 | 0.151 | 0.036*** | 0.031 | 0.052 |
| 103 | 0.196 | 0.064 | 0.002 | 0.006 | 0.028 | 0.041 | 0.026*** | 0.019 | 0.036 |
| 105 | 0.067 | 0.025 | 0.134 | 0.127 | 0.058 | 0.041 | 0.028** | 0.016 | 0.064 |
| 107 | 0.144 | 0.171 | 0.628 | 0.315 | 0.674 | 0.498 | 0.121** | 0.152 | 0.206 |
| 109 | 0.567 | 0.436 | 0.272 | 0.408 | 0.217 | 0.210 | 0.077** | 0.118 | 0.147 |
| 116 | 0.121 | 0.029 | 0.098 | 0.103 | 0.228 | 0.341 | 0.051*** | 0.063 | 0.086 |
| 120 | 0.152 | 0.056 | 0.114 | 0.047 | 0.047 | 0.049 | 0.0239*** | 0.029 | 0.040 |
| 134 | 0.026 | 0.000 | 0.510 | 0.562 | 0.319 | 0.025 | 0.150** | 0.143 | 0.225 |
| 138 | 0.192 | 0.115 | 0.007 | 0.027 | 0.186 | 0.095 | 0.039** | 0.042 | 0.061 |
| 148 | 0.167 | 0.134 | 0.057 | 0.088 | 0.190 | 0.190 | 0.028*** | 0.029 | 0.043 |
| 153 | 0.200 | 0.156 | 0.075 | 0.057 | 0.151 | 0.115 | 0.027*** | 0.031 | 0.044 |
| 160 | 0.271 | 0.079 | 0.033 | 0.016 | 0.013 | 0.024 | 0.049** | 0.055 | 0.079 |
| 162 | 0.099 | 0.110 | 0.091 | 0.125 | 0.513 | 0.267 | 0.084** | 0.063 | 0.115 |
| 168 | 0.040 | 0.020 | 0.037 | 0.053 | 0.176 | 0.033 | 0.023** | 0.015** | 0.031** |
| 169 | 0.008 | 0.004 | 0.144 | 0.379 | 0.008 | 0.004 | 0.060*** | 0.048 | 0.084 |
| 170 | 0.240 | 0.040 | 0.121 | 0.071 | 0.332 | 0.162 | 0.452** | 0.081 | 0.097 |
| 188 | 0.075 | 0.136 | 0.037 | 0.027 | 0.263 | 0.248 | 0.048*** | 0.053 | 0.077 |
| 189 | 0.138 | 0.185 | 0.023 | 0.023 | 0.060 | 0.084 | 0.030*** | 0.038 | 0.051 |
| 191 | 0.040 | 0.000 | 0.000 | 0.038 | 0.072 | 0.213 | 0.039** | 0.030 | 0.054 |
| 222 | 0.054 | 0.052 | 0.048 | 0.032 | 0.164 | 0.100 | 0.023*** | 0.015 | 0.031 |
| 224 | 0.024 | 0.065 | 0.024 | 0.020 | 0.042 | 0.083 | 0.011** | 0.017 | 0.022 |
| 227 | 0.003 | 0.010 | 0.170 | 0.174 | 0.208 | 0.081 | 0.044** | 0.041 | 0.065 |
| 268 | 0.242 | 0.082 | 0.051 | 0.067 | 0.059 | 0.037 | 0.028*** | 0.020 | 0.038 |
| 281 | 0.097 | 0.080 | 0.008 | 0.014 | 0.074 | 0.049 | 0.022** | 0.020 | 0.032 |
| 300 | 0.069 | 0.085 | 0.028 | 0.024 | 0.037 | 0.017 | 0.016** | 0.018 | 0.026 |
| 350 | 0.000 | 0.000 | 0.109 | 0.301 | 0.000 | 0.000 | 0.069** | 0.060 | 0.100 |
| 396 | 0.101 | 0.063 | 0.012 | 0.040 | 0.006 | 0.010 | 0.023*** | 0.021 | 0.034 |
| 409 | 0.018 | 0.003 | 0.082 | 0.130 | 0.180 | 0.249 | 0.044*** | 0.041 | 0.065 |
| 413 | 0.029 | 0.070 | 0.153 | 0.384 | 0.017 | 0.014 | 0.048*** | 0.048 | 0.073 |
| 480 | 0.104 | 0.088 | 0.020 | 0.030 | 0.035 | 0.030 | 0.020** | 0.022 | 0.032 |
| 531 | 0.380 | 0.233 | 0.151 | 0.083 | 0.163 | 0.113 | 0.057** | 0.042 | 0.078 |
| 561 | 0.014 | 0.037 | 0.051 | 0.014 | 0.103 | 0.032 | 0.013** | 0.020 | 0.025** |
| 570 | 0.087 | 0.134 | 0.038 | 0.040 | 0.027 | 0.083 | 0.022** | 0.022 | 0.034 |
| 668 | 0.029 | 0.059 | 0.034 | 0.124 | 0.005 | 0.038 | 0.017** | 0.033 | 0.038 |
| 832 | 0.021 | 0.013 | 0.064 | 0.003 | 0.157 | 0.038 | 0.022** | 0.015*** | 0.029 |
| 846 | 0.002 | 0.002 | 0.092 | 0.139 | 0.161 | 0.172 | 0.046** | 0.038 | 0.065 |
| 993 | 0.129 | 0.088 | 0.007 | 0.020 | 0.028 | 0.022 | 0.031** | 0.030 | 0.046 |
| 1089 | 0.044 | 0.091 | 0.027 | 0.020 | 0.128 | 0.055 | 0.017*** | 0.019 | 0.027** |
| 1168 | 0.051 | 0.147 | 0.046 | 0.015 | 0.008 | 0.015 | 0.025** | 0.020 | 0.036 |
| 1353 | 0.024 | 0.011 | 0.140 | 0.021 | 0.033 | 0.019 | 0.018** | 0.020 | 0.02912** |
