# Supplementary material for: Characterisation of the Faecal Bacterial Community in Adult and Elderly Horses Fed a High Fibre, High Oil or High Starch Diet Using 454 Pyrosequencing
Source: PLoS One. 2014 Feb 4;9(2):e87424. doi: 10.1371/journal.pone.0087424 (PMC3913607; doi:10.1371/journal.pone.0087424)
Supplement: Table S5 — Classification of each OTU significant (P<0.001) for Diet, Age or Diet*Age. (DOCX) [file pone.0087424.s007.docx]

**Table S5** Classification of each OTU significant (P<0.001) for Diet, Age or Diet*Age

| **OTU** | **Phyla** | **Class** | **Order** | **Family** | **Genus** |
| --- | --- | --- | --- | --- | --- |
| 6 | Firmicutes | Clostridia | Clostridiales | Lachnospiraceae | Unclassified |
| 8 | Bacteroidetes | Bacteroidia | Bacteroidales | Prevotellaceae | Paraprevotella |
| 11 | Firmicutes | Clostridia | Clostridiales | Lachnospiraceae | Unclassified |
| 18 | Firmicutes | Clostridia | Clostridiales | Lachnospiraceae | Roseburia |
| 22 | Firmicutes | Clostridia | Clostridiales | Lachnospiraceae | Unclassified |
| 26 | Proteobacteria | Gammaproteobacteria | Aeromonadales | Succinivibrionaceae | Succinivibrio |
| 27 | Bacteroidetes | Bacteroidia | Bacteroidales | Bacteroidales_incertae_sedis | Phocaeicola |
| 31 | Firmicutes | Clostridia | Clostridiales | Lachnospiraceae | Unclassified |
| 36 | Firmicutes | Clostridia | Clostridiales | Lachnospiraceae | Unclassified |
| 62 | Firmicutes | Clostridia | Clostridiales | Lachnospiraceae | Unclassified |
| 66 | Bacteroidetes | Bacteroidia | Bacteroidales | Rikenellaceae | Rikenella |
| 68 | Firmicutes | Clostridia | Clostridiales | Lachnospiraceae | Unclassified |
| 90 | Firmicutes | Negativicutes | Selenomonadales | Acidaminococcaceae | Acidaminococcus |
| 103 | Firmicutes | Clostridia | Clostridiales | Ruminococcaceae | Pseudoflavonifractor |
| 105 | Firmicutes | Clostridia | Clostridiales | Lachnospiraceae | Unclassified |
| 107 | Firmicutes | Clostridia | Clostridiales | Lachnospiraceae | Lachnospiracea_incertae_sedis |
| 109 | Unclassified | Unclassified | Unclassified | Unclassified | Unclassified |
| 116 | Firmicutes | Clostridia | Clostridiales | Lachnospiraceae | Unclassified |
| 120 | Firmicutes | Clostridia | Clostridiales | Lachnospiraceae | Unclassified |
| 134 | Firmicutes | Clostridia | Clostridiales | Ruminococcaceae | Oscillibacter |
| 138 | Firmicutes | Unclassified | Unclassified | Unclassified | Unclassified |
| 148 | Firmicutes | Clostridia | Clostridiales | Unclassified | Unclassified |
| 153 | Firmicutes | Clostridia | Clostridiales | Lachnospiraceae | Unclassified |
| 160 | Proteobacteria | Alphaproteobacteria | Unclassified | Unclassified | Unclassified |
| 162 | Firmicutes | Clostridia | Clostridiales | Ruminococcaceae | Unclassified |
| 168 | Firmicutes | Clostridia | Clostridiales | Clostridiales_Incertae Sedis XIII | Mogibacterium |
| 169 | Proteobacteria | Gammaproteobacteria | Aeromonadales | Succinivibrionaceae | Unclassified |
| 170 | Bacteroidetes | Bacteroidia | Bacteroidales | Prevotellaceae | Paraprevotella |
| 188 | Firmicutes | Clostridia | Clostridiales | Lachnospiraceae | Lachnospiracea_incertae_sedis |
| 189 | Firmicutes | Clostridia | Clostridiales | Lachnospiraceae | Unclassified |
| 191 | Firmicutes | Clostridia | Clostridiales | Lachnospiraceae | Unclassified |
| 222 | Firmicutes | Clostridia | Clostridiales | Ruminococcaceae | Flavonifractor |
| 224 | Firmicutes | Clostridia | Clostridiales | Lachnospiraceae | Pseudobutyrivibrio |
| 227 | Firmicutes | Negativicutes | Selenomonadales | Acidaminococcaceae | Unclassified |
| 268 | Spirochaetes | Spirochaetes | Spirochaetales | Spirochaetaceae | Treponema |
| 281 | Actinobacteria | Actinobacteria | Coriobacteridae | Coriobacteriales | Coriobacterineae |
| 300 | Firmicutes | Clostridia | Clostridiales | Lachnospiraceae | Unclassified |
| 350 | Firmicutes | Clostridia | Clostridiales | Clostridiaceae 1 | Clostridium sensu stricto |
| 396 | Firmicutes | Unclassified | Unclassified | Unclassified | Unclassified |
| 409 | Firmicutes | Clostridia | Clostridiales | Ruminococcaceae | Oscillibacter |
| 413 | Firmicutes | Clostridia | Clostridiales | Clostridiaceae 1 | Clostridium sensu stricto |
| 480 | Unclassified | Unclassified | Unclassified | Unclassified | Unclassified |
| 531 | Unclassified | Unclassified | Unclassified | Unclassified | Unclassified |
| 561 | Firmicutes | Clostridia | Clostridiales | Lachnospiraceae | Unclassified |
| 570 | Firmicutes | Clostridia | Clostridiales | Lachnospiraceae | Unclassified |
| 668 | Bacteroidetes | Unclassified | Unclassified | Unclassified | Unclassified |
| 832 | Firmicutes | Clostridia | Clostridiales | Lachnospiraceae | Unclassified |
| 846 | Firmicutes | Clostridia | Clostridiales | Ruminococcaceae | Oscillibacter |
| 993 | Firmicutes | Unclassified | Unclassified | Unclassified | Unclassified |
| 1089 | Actinobacteria | Actinobacteria | Coriobacteridae | Coriobacteriales | Unclassified |
| 1168 | Firmicutes | Clostridia | Clostridiales | Lachnospiraceae | Unclassified |
| 1353 | Bacteroidetes | Bacteroidia | Bacteroidales | Unclassified | Unclassified |
