# Supplementary material for: Characterisation of the Faecal Bacterial Community in Adult and Elderly Horses Fed a High Fibre, High Oil or High Starch Diet Using 454 Pyrosequencing
Source: PLoS One. 2014 Feb 4;9(2):e87424. doi: 10.1371/journal.pone.0087424 (PMC3913607; doi:10.1371/journal.pone.0087424)
Supplement: Table S6 — Classification of the core bacterial community in the faeces of horses fed three different diets. (DOCX) [file pone.0087424.s008.docx]

**Table S6**. Classification of the core bacterial community in the faeces of horses fed three different diets.

|  | **O.T.U.** | **Phyla** | **Class** | **Order** | **Family** | **Relative Abundance (%)** | **Standard deviation** |
| --- | --- | --- | --- | --- | --- | --- | --- |
| **ALL** | 375 | Firmicutes | Clostridia | Clostridiales | Ruminococcaceae | 0.25 | 0.003 |
|  | 237 | Bacteroidetes | Unclassified | Unclassified | Unclassified | 0.27 | 0.003 |
|  | 15,38 | Firmicutes | Clostridia | Clostridiales | Clostridiales_Incertae Sedis XIII | 0.50 | 0.005 |
|  | 6, 78 | Firmicutes | Clostridia | Clostridiales | Lachnospiraceae | 1.29 | 0.012 |
| **HAY** | 375 | Firmicutes | Clostridia | Clostridiales | Ruminococcaceae | 0.24 | 0.008 |
|  | 221 | Bacteroidetes | Bacteroidia | Bacteroidales | Unclassified | 0.25 | 0.012 |
|  | 531 | Unclassified | Unclassified | Unclassified | Unclassified | 0.28 | 0.014 |
|  | 66 | Bacteroidetes | Bacteroidia | Bacteroidales | Rikenellaceae | 0.45 | 0.026 |
|  | 15,38 | Firmicutes | Clostridia | Clostridiales | Clostridiales_Incertae Sedis XIII | 0.53 | 0.021 |
|  | 21,82 | Firmicutes | Erysipelotrichia | Erysipelotrichales | Erysipelotrichaceae | 0.58 | 0.017 |
|  | 3 | Firmicutes | Bacilli | Lactobacillales | Lactobacillaceae | 0.65 | 0.027 |
|  | 92,237 | Bacteroidetes | Unclassified | Unclassified | Unclassified | 0.90 | 0.038 |
|  | 8 | Bacteroidetes | Bacteroidia | Bacteroidales | Prevotellaceae | 1.13 | 0.049 |
|  | 5 | Firmicutes | Clostridia | Unclassified | Unclassified | 1.20 | 0.044 |
|  | 7 | Fibrobacteres | Fibrobacteria | Fibrobacterales | Fibrobacteraceae | 1.25 | 0.070 |
|  | 4,9,13,96 | Bacteroidetes | Bacteroidia | Bacteroidales | Porphyromonadaceae | 3.21 | 0.107 |
|  | 6,10,11,22,25,29,36,78,232,262,949,1047 | Firmicutes | Clostridia | Clostridiales | Lachnospiraceae | 5.26 | 0.109 |
| **CHO** | 1414 | Bacteroidetes | Bacteroidia | Bacteroidales | Porphyromonadaceae | 0.17 | 0.006 |
|  | 6 | Firmicutes | Clostridia | Clostridiales | Unclassified | 0.21 | 0.038 |
|  | 47 | Firmicutes | Clostridia | Clostridiales | Clostridiaceae 1 | 0.26 | 0.008 |
|  | 411 | Firmicutes | Clostridia | Clostridiales | Ruminococcaceae | 0.30 | 0.026 |
|  | 96 | Bacteroidetes | Unclassified | Unclassified | Unclassified | 0.32 | 0.008 |
|  | 3 | Unclassified | Unclassified | Unclassified | Unclassified | 0.33 | 0.038 |
|  | 38,413 | Firmicutes | Clostridia | Clostridiales | Clostridiales_Incertae Sedis XIII | 0.44 | 0.016 |
|  | 78 | Firmicutes | Erysipelotrichia | Erysipelotrichales | Erysipelotrichaceae | 0.47 | 0.023 |
|  | 15 | Firmicutes | Bacilli | Lactobacillales | Lactobacillaceae | 0.75 | 0.004 |
|  | 18,21,109,237,375 | Firmicutes | Clostridia | Clostridiales | Lachnospiraceae | 2.17 | 0.027 |
| **FAT** | 20 | Spirochaetes | Spirochaetes | Spirochaetales | Spirochaetaceae | 0.37 | 0.023 |
|  | 109,531 | Unclassified | Unclassified | Unclassified | Unclassified | 0.37 | 0.014 |
|  | 122,375 | Firmicutes | Clostridia | Clostridiales | Ruminococcaceae | 0.40 | 0.010 |
|  | 15,38 | Firmicutes | Clostridia | Clostridiales | Clostridiales_Incertae Sedis XIII | 0.52 | 0.014 |
|  | 63 | Proteobacteria | Alphaproteobacteria | Rhizobiales | Unclassified | 0.53 | 0.053 |
|  | 145,365 | Bacteroidetes | Bacteroidia | Bacteroidales | Unclassified | 0.57 | 0.021 |
|  | 17,92,237 | Bacteroidetes | Unclassified | Unclassified | Unclassified | 1.03 | 0.031 |
|  | 5 | Firmicutes | Unclassified | Unclassified | Unclassified | 1.17 | 0.082 |
|  | 4 | Bacteroidetes | Bacteroidia | Bacteroidales | Porphyromonadaceae | 1.40 | 0.078 |
|  | 6,11,25,29,36,37,78,280,411,1047 | Firmicutes | Clostridia | Clostridiales | Lachnospiraceae | 3.96 | 0.079 |
